# Public sector reform and demand for human resources for health (HRH)

**DOI:** 10.1186/1478-4491-2-15

**Published:** 2004-11-23

**Authors:** Jane Lethbridge

**Affiliations:** 1 Independent health policy consultant, London, UK

## Abstract

This article considers some of the effects of health sector reform on human resources for health (HRH) in developing countries and countries in transition by examining the effect of fiscal reform and the introduction of decentralisation and market mechanisms to the health sector.

Fiscal reform results in pressure to measure the staff outputs of the health sector. Financial decentralisation often leads to hospitals becoming "corporatised" institutions, operating with business principles but remaining in the public sector. The introduction of market mechanisms often involves the formation of an internal market within the health sector and market testing of different functions with the private sector. This has immediate implications for the employment of health workers in the public sector, because the public sector may reduce its workforce if services are purchased from other sectors or may introduce more short-term and temporary employment contracts.

Decentralisation of budgets and administrative functions can affect the health sector, often in negative ways, by reducing resources available and confusing lines of accountability for health workers. Governance and regulation of health care, when delivered by both public and private providers, require new systems of regulation.

The increase in private sector provision has led health workers to move to the private sector. For those remaining in the public sector, there are often worsening working conditions, a lack of employment security and dismantling of collective bargaining agreements.

Human resource development is gradually being recognised as crucial to future reforms and the formulation of health policy. New information systems at local and regional level will be needed to collect data on human resources. New employment arrangements, strengthening organisational culture, training and continuing education will also be needed.

## Introduction

This paper considers health sector reform and its impact on human resources for health (HRH) in developing countries and countries in transition. Health sector reform has been defined as the "sustained purposeful change to improve the efficiency, equity and effectiveness of the health sector" [1]. Health sector reform involves many fundamental changes to the way in which public services are financed, organised and delivered in both developing and developed countries, and often operates as part of a wider programme of public sector reform. Fiscal reform, the introduction of market mechanisms and decentralisation are three key elements of health sector reform. This paper will show the impact of these elements on human resources for health and attempt to assess the changing demand for health workers. A series of recommendations will seek to address some of the issues that have emerged for HRH demand during the process of health sector reform.

## Impact of health sector reform on human resources for health (HRH)

### Fiscal reform

The introduction of new budget management systems, designed to maintain financial control throughout government, is one of the most important elements of fiscal reform. These incorporate new financial planning and control systems that emphasise what outputs a department or agency will be expected to deliver. Overall, there is a focus on the performance of public services [2]. Mechanisms for monitoring and enforcement of targets are designed for all government departments. In the health sector, over 50% of costs are labour costs, so that demonstrating effectiveness depends largely on attempting to measure the work of health staff. Measuring outputs in health care is often difficult because of having to capture both the quality of care and patient outcomes.

Fiscal reform introduces new ways of allocating resources in line with government objectives [3]. There may not be a precise match with individual sectoral objectives. Fiscal reform also tries to encourage improved use of resources, which may inform the reorganisation and management of central agencies and the downsizing of the civil service.

Health sector employees are often part of the civil service, and so civil service reform has an impact on the employment and deployment of health workers. Civil service downsizing results from policies to cut the costs of the public sector and transfer the delivery of services to the private or non-profit sectors. These changes lead to a reduction in the size of the public health care workforce. Compensation schemes may include retraining and lump-sum severances to ease the transition of workers into the private sector. This may be accompanied by wage policy reform to limit and contain wage expenditures, again with the potential to affect health workers [4].

Trying to improve the performance of the health sector, one of the objectives of health sector reform, has been a slow process because the savings from reducing the size of the workforce are often not enough to raise salaries for the remaining staff. Several countries, including Zambia, have set up a separate health service agency, operating as a semi-autonomous government agency, that employs staff directly. Some writers argue that agencies need to be well-managed on a limited budget rather than be seen as an escape from civil service restrictions [5]. In addition, "the importance of political and institutional context in which reforms have to be implemented has been undervalued" [5]. Many ministries have a poor record of human resource management and planning. New information systems can provide a more accurate picture of the current number, type and distribution of staff, but civil service systems rarely provide incentives to reduce staff budgets. There may also be attempts to strengthen linkages between government departments, which are relevant for the health sector.

The decentralisation of budget management is another element of fiscal reform. In the health sector, this has been reflected in the decentralisation of service provision to semi-autonomous hospitals, because hospitals often consume the largest part of the health sector budget [6]. Set up as institutions run on business principles, "corporatised" hospitals bring the results of fiscal reform to local level. With limited available resources, there may be pressure to generate income through the introduction of user fees such as for health services, as well as trying to achieve outputs and outcomes at the lowest cost. Delegation of financial authority can also provide managers with the scope to use existing resources differently or consider different ways of delivering services, such as by using a range of local providers [2].

The motivations for introducing corporatisation may also vary from wanting to increase efficiency and achieve cost saving and quality improvements to just wanting to "free up a public function from constraints of ... red tape" [6]. Cassels argues that decentralisation has provided "major contradictions for health care" between accountability, competing priorities and equity and tensions between small-scale participation and managerial effectiveness required at a large scale [5].

In Mexico, new systems of financial management affected public health institutions by restricting the maintenance and upgrading of equipment and imposing cuts in the wages of health workers. This has led to the deterioration of working conditions and the quality of care provided by the public health sector [7].

### Financial management

Part of a programme of fiscal reform involves the development of new systems and structures of financial management, which have organisational implications [3]. The role and functions of the finance ministry in central government are strengthened and it develops a dominant role over other government departments. This affects government health ministries, because the priorities of the finance ministry are often different from those of the health ministry and priority setting and resource allocation issues become sources of conflict. The nature of the relationship between the finance and health ministries has been exposed in the development of poverty reduction strategy papers [8].

In health systems, new forms of financing for the health sector may involve moving from a tax-based system to an insurance system. This, in turn, introduces new forms of budgetary management and control between insurance funds and health service providers as well as new systems of payment collection. Financial management is often accompanied by new information technology systems, which have the potential to change the ways in which public services are monitored [2].

### Market mechanisms

The introduction of market mechanisms is often driven by the goal of fiscal stability. Stronger systems of budget and management control are introduced that focus on results. They affect sectoral priorities and available human resources. Market mechanisms may be introduced by making health care institutions operate within an internal health care market and subjecting some health services to wider market testing.

As part of developing a managed market in the public sector, the health sector is often reorganised into two separate purchasing and provider functions. The purchasing entity, typically a national or regional health authority, buys services from provider units within the government sector and is also encouraged to buy services from a range of providers in the private and NGO sectors [9]. This has immediate implications for the employment of health workers in the public sector, because the public sector may reduce its workforce if services are purchased from other sectors. The private sector may start to expand. Health workers often move from the public to the private sector because of better prospects and higher pay. Market testing has often led to changes in the size of the public sector workforce, increasing short-term and temporary employment contracts, and changes in wage levels [10,11].

### User fees

User charges have been introduced as a way of generating income for the health sector. User fees in many countries have affected access to services and equity [12,13]. In Nicaragua, the introduction of user fees and separate services for private, paying patients started as a national initiative but is now incorporated into local health systems. User fees have become the main source of decentralised revenue. At hospital level, 30% goes towards salary supplements [14]. In Honduras, the revenues collected from user fees have contributed only 2% to the Ministry of Health expenditures but the administrative costs are 67% of the revenues collected [15]. In most countries, however, the preparation of staff and supporting systems for implementing user charges has been minimal. The introduction of user fees places new pressures on health workers, especially when user fees contribute to the actual wages and salaries of health workers.

A recent World Bank report (2002) presents informal payments as a hindrance to health sector reform. Payments made to health workers are considered to draw resources away from the health care system because they are given to individuals rather than institutions. Such payments operate as a private, unregulated system and the practice is often illegal. Poor people often avoid using health care facilities because of the need to make informal payments [16]. Stronger management capacity is needed to support and coordinate public, private and NGO providers and provide accountability so that revenue from user fees goes directly for service improvements [17]. This would also depend on health workers' being paid an adequate salary and the introduction of transparent systems to support the collection of user fees within the health care sector.

### Performance management

Public sector and health sector reform often introduce new approaches to managing staff. Perhaps the most important innovation is "thinking differently about staff", which effectively underpins other changes. The three most innovative dimensions are "flexible staffing and recruitment practices, recognising achievement and developing performance contracts" [2].

The element of fiscal reform that emphasises outputs and outcomes of government services informs the development of performance management. It aims to address management problems relating to poor employee performance management, wage and non-wage incentives, job classification systems and ineffective payroll and personnel systems. Performance management may also be introduced as a way of improving standards within public services and making services more responsive to citizens. Wider programmes of training and capacity building for staff can accompany this. Some developing countries have experimented with performance management systems, with limited success [9]. Often the new "corporatised" hospitals have only limited management autonomy, and governments lack the capacity to manage performance in the health system [9].

### Decentralisation

The delegation and decentralisation of administrative and management processes often accompany budgetary reforms. In Nicaragua, decentralisation was used to introduce market reforms. Budget cuts, loss of resources from primary health care, user fees and privatisation were introduced at the same time [14]. In 1991 Local Integrated Health Care Systems (SILAIS) were introduced, which are made up of a hospital and a network of primary care units. Each SILAIS has a separate Board of Directors consisting of local officials, church officials, health sector representatives, community members and the SILAIS director. This group monitors services and approves the local health plan and budget, but accountability remains unclear. The Ministry of Health controls funding through "performance agreements, and controls 80% of the health budget including staff levels and composition". Only recently have Local Health Systems been given the power to sack staff [14].

In Uganda at the time of decentralisation, salaries for staff on the payroll were a central responsibility, although this has now been decentralised through a special conditional grant. In the past, professional staff were put on the national payroll and nursing aides were hired locally for work in rural health centres and health posts and paid for by the Ministry of Local Government. After the decentralisation reforms, nursing aides were supposed to be paid by local committees, but in practice this often did not happen and they were not paid for long periods [18].

Botswana and Tanzania have had long experience of decentralisation. As a result of health sector reform, health staff were transferred to local government contracts although senior staff remained employed by the Ministry of Health. This has led to confused loyalties and management responsibilities. In some districts the "personality factor" has meant that individuals working together have managed to overcome some of these problems, in spite of the systems introduced. Senior staff who have subsequently been transferred to local government complain that that there is "little relationship between promotions/disciplinary actions and performance". In both countries there is some scope for local decision-making in relation to personnel management, but there is still resistance to distributing staff according to local needs. More incentives and other measures are considered necessary if regional imbalances of staff are to be addressed [17].

Decentralisation may lead to a loss of resources for the health sector. In Uganda, after decentralisation, once central government stopped a block grant, primary heath care was not given the allocation at local level that had been expected by the Ministry of Finance. There were also considerable district variations in the allocation of health resources. Although some districts did increase their health allocation, in many cases decentralisation led to fewer resources for health. One of the reasons cited for the decline in allocation of resources to the health sector was that a large part of the health budget goes on salaries and wages, which do not show any dramatic change in the sector. Decentralisation in this context led to problems of financial management and corruption at local level, new problems of governance with a lack of accountability and concerns over quality of services [18].

Some changes have run contrary to the main aims of reform, such as increased centralisation of controls over pay. Much health sector reform was to strengthen and rationalise budgeting, financial control and staff classification, but in some cases control over health sector staffing has remained at national level [19]. Even when transfer of budgets has taken place, there is confusion between local government and health sector responsibilities.

### Changes in provision

The use of the private sector as a health service provider has had implications for the recruitment and retention of staff in the public sector. Some services have been privatised and are now run by local, national or international private companies. Other services have been contracted out to both private and non-profit service providers. This has resulted in movement of health workers from public to private or non-profit sectors [17].

In the public institutions that remain, market conditions have been introduced and services are contracted out, which has resulted in a widespread decrease in job security in many countries. Health workers have moved from collective-bargaining arrangements to individual contracts. Decentralisation and privatisation have contributed to the breakdown of national collective bargaining. In Eastern and Central Europe, new organisations and professional associations and reorganised trade unions have led to a breakdown in labour relations expertise [20].

Changes of responsibility for managing health services, from national to local level and from public to private sectors, have led to some confused accountabilities for health workers [11]. Health workers have moved from being accountable to both a public service and to their profession, to being accountable to a commercial employer with performance-related pay and conditions. This often causes tension between professional standards and pressure from the commercial employer.

The process of health sector reform has had an impact on human resources for health through new systems of financial and performance management, decentralisation and the introduction of market mechanisms. This has led to changes in the demand for health workers and in some cases the types of skills and expertise required from health workers. At the same time, the capacity of the new management systems is unable to create conditions in which a new health workforce can be developed. This can be seen particularly clearly in the process of budget decentralisation, which leads to a focus on local decision-making but where the capacity of local institutions to recruit, train and manage local health service workers is limited. This has an influence on the quality of health care delivered.

## Implications for HRH demand

Demand for human resources in health systems that have experienced health sector reform must be considered in terms of the numbers of health workers and the skills and expertise needed currently and what will be anticipated in the future. There is a growing awareness that human resource issues need to be prioritised more effectively within reforms in order to secure an adequate health care workforce to deliver services now and in the future.

### Public sector culture

Although health sector reform has included elements of human resources strategies such as improved education and training, restructured salary scales and a closer link between performance and reward, it has also had a fundamental impact on organisational culture and public sector ethos, which in turn influence demand for human resources.

A study of four countries in Eastern and Southern Africa concluded that "human resource development, personnel management and staff motivation are critical issues" [17]. Tanzania, although it has invested in human resources development, found that low salaries, delayed promotion opportunities and poor working conditions led to dissatisfaction in the workforce. Staff performance has been found to be unsatisfactory. Although monetary and non-monetary allowances were supposed to compensate for low wages, they have led to poor teamwork and lack of continuity in health service operations. The regional health team was found to spend 40% of its time out of the region on training and meetings.

Burkina Faso introduced health sector reforms in 1991 but they have not been fully implemented. It has recently introduced civil service reform, which "aims at a more flexible management and better performances of personnel". In a country where services are centralised, with an imbalance in personnel and low staff motivation and poor standards of care, there is resistance to the new reform. There has been a decline in standards of service between 1986 and 1997 [21]. Poor financial and human resources policies and management are resulting in high cost and poor quality of care. A recent study concluded: "Human resources should become the central focus for reform" [21].

Matheson (2002) points out that "the least systemically orientated area of recent public management reforms has been human resource management.... There is a danger that the constitutional, legal, cultural and leadership factors, which together create what is important and distinctive about public services, are not reflected on, or are dismissed as the bureaucratic problem which must be 'reformed' " [22].

### Demand for health workers

Some national health sector reforms reduced the numbers employed in the health sector, as in Chile and Latvia. Others, as in Mexico and Zambia, led to a rise in employment [11]. In almost all countries, health worker employment was restructured. Health sector reform has often aimed to restructure organisations to reduce costs and the power of the workforce. Pressure of work and hours worked have in many cases increased since health sector reform. There is also an increased workload due to lack of staff, pressure for results and staff reductions [10,11]. This affects the future demand for health workers.

Health services have usually been seen as "essential services" and so health workers had the legal status of public servants. They had to account to both employers and professional bodies subject to strict regulation and registration rules. The effect of privatisation has been to change the pay and terms of employment and the legal status of health workers [11]. The public health sector has changed from being a public service to one with a greater commercial focus. This may have an effect on recruitment. The Nicaraguan government has continued to cut the number of doctors, changing to an hourly rate of reimbursement rather than salaries, and ending the commitment of government to employ graduating medical students [14]. This process is effectively influencing the demand for doctors in the public sector.

The introduction of flexible contracts and fall in full-time permanent contracts has been a characteristic of most reforms, leading to a reduction in long-term employment security. Some of these changes in terms and conditions have led to health workers' taking on second jobs. This may be caused by the increase in part-time employment and low and erratically paid wages [20]. In Eastern and Central Europe, women have been most affected by the reduction in jobs in the heath sector. Their prospects for redeployment are often limited due to a lack of mobility [11]. The growth of part-time work in the public sector is a sign of the changing demand for health workers.

Low pay levels have led to staff leaving the public sector and moving to the private sector, NGOs and aid agencies [9]. Low pay also contributes to low administrative capacity, as well as poor organisational discipline. In an analysis of health worker motivation, health sector reform was found to influence health worker motivation through changing organisational structures and community-client roles [23]. Organisational factors influence worker motivation through management structures and processes, communication processes, organisational support structures and processes, and ways of providing feedback about organisational and individual performance.

These changes in organisational culture have often had a negative impact on workers' motivation. Important informal factors – for example, staff commitment – have "become the prime means of direction, motivation, coordination and control" [22]. When staff commitment deteriorates over time, health workers may migrate, not only from the public sector to the private sector, but internationally. This results in a shortage of skilled health workers within the public sector, precipitating a growing demand for skilled health workers.

The aim of introducing market mechanisms to the public sector has been to improve economic efficiency. New skills are needed to implement commissioning and contracting of services. For example, contracts can be a powerful form of regulation if drawn up and monitored effectively, but increased expertise is required to establish this form of regulation.

### Process of reform

Understanding the process of reform is important for understanding how changes have taken place but also what the critical factors are for successful policy implementation in future. "The process of reform offers numerous opportunities to alter the political equations that impede change" [19]. This is also significant for understanding the potential role that health workers can play within reforms.

In Latin America, health sector reform has been characterised by various forms of privatisation, competition among providers, new insurance systems, management autonomy for hospitals and increased community participation. The goals were efficiency, accountability and improved quality of services. A recent study looked at how groups, such as unions, play different roles in relation to reforms with some opposing and others supporting reform. Some public sector health workers have played an important role in supporting change [24]. The importance of "principled agents" in public sector organisations has also been noted [25]. Public servants can be motivated by managerial and incentive schemes to lead and support change. Linking popular and unpopular reforms has often led to reformers' changing their attitudes to reform. Networks of reformers can also play a role in supporting reformers in environments hostile to change [19].

Communities also influence health worker motivation through their expectations of services. As health sector reform also aims to empower service users, this focus will have a significant impact on the individual health worker in future [23]. The evidence showing the extent to which users of health services have been empowered by health sector reform is limited in developing countries [13].

However, health workers do play an important role in the implementation of health sector reform policies. The lack of consideration of the value of human resources in health sector reform programmes has meant that this has often been ignored. Gilson et al. (2003) recommended that: "Technical analysts might consider working with middle managers and health workers to ensure adequate consideration is given to implementation realities in proposal development" [12].

### Knowledge gaps

The process of health sector reform is not complete. More research is needed to monitor changes still taking place as well as the outcomes of the reforms. One of the major changes is the role that health workers play in both the public and private sectors. How health workers perceive their roles in the different sectors and what the implications are for motivation, particularly in the public sector, will need further exploration.

The role of the public sector is changing and this is reflected in public sector institutions and the public sector ethos. How this changed public sector can demonstrate a commitment to health workers as well as harnessing their own commitment, still needs to be explored.

A further area of research needs to improve the understanding of human resources in health sector appraisal studies "by incorporating functional, institutional and policy dimensions. Only then will human resources become in practice the most valuable resource within any national system" [26].

## Conclusion

There is a growing awareness that human resources for health (HRH) must be addressed more effectively within public sector reform. Stein thinks that HRH strategies need to be a "primary objective for public organisations" [27].

Public sector reforms have sometimes been characterised as containing the paradox of aiming to reward performance and empower staff whilst at the same time implementing downsizing and redundancy – the "human costs of reform" [28]. Issues resulting from these changes include loss of institutional memory and the use of downsizing as a way of making financial savings rather than administrative reform. Changing rules and processes do not always lead to changes in organisational culture. "Multi-faced interventions sustained long enough to achieve change" will be needed to change public sector culture [13].

To address some of these issues, action at strategic, regional and local levels will be needed to strengthen skills, expertise and analysis of HRH and to strengthen the integration of HRH issues into health policy making and with relevant agencies [26]. New human resources systems are needed at regional and local level.

Additional data on existing employment, retention and deployment issues must be made available to decision makers so as to relate them to health equity issues [29]. An increased awareness of the importance of improved coordination of facility planning and human resource planning at national and local level is needed. A better understanding of the role of organisational culture and public sector ethos in health worker motivation is needed [23]. This might be achieved by developing case studies of health workers as "drivers of change" and more process research to look at emerging practice.

The working conditions of health workers need to be improved. This might be achieved through developing more flexible employment arrangements that are employee-focused. The public sector needs to be encouraged to establish a "living wage" and other forms of worker security so that terms and conditions of public sector workers are better than those of private sector workers [26]. Health workers need to have access to continuous professional development that includes skills for performance management, management of contracts and other new ways of operating in reformed systems [29].

The role of central government in setting standards for professional practice and legal requirements for registration needs to be strengthened so that human resources policies, registration and regulation are mutually supportive. Registration requirements that include experience in rural or remote areas would help to address uneven distribution of health workers.

## Declaration of competing interests

The author(s) declare that they have no competing interests.
